# Comparative effectiveness of two intravenous immunoglobulin products in children with Kawasaki disease, a nationwide cohort study

**DOI:** 10.1038/s41598-023-45092-5

**Published:** 2023-10-30

**Authors:** Ni-Chun Kuo, Ching-Heng Lin, Ming-Chih Lin

**Affiliations:** 1 Children’s Medical Center, Taichung Veterans General Hospital, 1650 Taiwan Boulevard Sec. 4, Taichung, 40705 Taiwan; 2 Department of Medical Research, Taichung Veterans General Hospital, Taichung, Taiwan; 3 Department of Post-Baccalaureate Medicine, College of Medicine, National Chung Hsing University, Taichung, Taiwan; 4 School of Medicine, National Yang Ming Chiao Tung University, Taipei, Taiwan; 5 Department of Food and Nutrition, Providence University, Taichung, Taiwan; 6 School of Medicine, Chung Shan Medical University, Taichung, Taiwan

**Keywords:** Cardiology, Medical research

## Abstract

Kawasaki Disease (KD) is the most common acquired pediatric heart disease in the developed world. Rapid infusion of high-dose intravenous immunoglobulin is the standard therapy. Different manufacturing processes of IVIG may influence their efficacy. This study aims to conduct a head to head comparison of two IVIGs, TBSF and Privigen, from a nationwide perspective. The main data source was the National Health Insurance Research Database (NHIRD) of Taiwan. A total of 3368 KD cases involving children under 2 years of age were enrolled from January 2015 to November 2020. The primary endpoint was IVIG resistance, which we defined as the total amount exceeding 26 g in one admission. The secondary endpoints encompassed two distinct criteria: coronary involvement, which was defined as the prolonged use of aspirin or anti-coagulation agents between 180 and 360 days after the index date, and recurrence, which was defined as readmission for IVIG therapy occurring more than 30 days after previous KD index day and continuing until the end of the follow-up period. Privigen demonstrated a lower IVIG resistance rate at 9.4% in comparison to TBSF, which exhibited a rate of 9.7% (odds ratio 0.72, 95% CI 0.52–0.99). Privigen had a lower odds of coronary involvement (odds ratio 0.38, 95% CI 0.18–0.82). There is no difference in recurrence rate (odds ratio 0.60, 95% CI 0.22–1.68). Privigen might have a lower rate of IVIG resistance and reduced coronary artery involvement. The discrepancy may be due to the concentration, the stabilizers, or the source of plasma. Further investigation is needed to compare the effectiveness of different IVIGs in the large randomized controlled clinical trial.

## Introduction

Kawasaki Disease (KD) is an acute form of multi-systemic vasculitis, characterized by prolonged fever and systemic principal features including conjunctivitis, cervical lymphadenopathy, changes in extremities, polymorphous skin rashes, and changes in oral mucosa. KD occurs predominantly among preschool children and is capable of causing acquired pediatric cardiac disease, ranging from acute phase coronary aneurysms to long-term sequelae, such as coronary stenosis and obstruction^1,2^. For the acute stage of KD, most physicians follow the standard protocol of the American Academy of Pediatrics (AAP) and American Heart Association (AHA), which includes rapid infusion of intravenous immunoglobulin (IVIG) (2 g/kg) in 12 h along with oral aspirin^3–5^. As the most crucial medication in the acute stage of KD, IVIG was reported to decrease the rate of coronary aneurysms from 20 to 25% to less than 5%^6,7^. IVIG, precipitated immunoglobulin G (IgG) from the plasma of thousands of blood donors, provides an immunomodulatory effect and suppresses inflammation by binding the heavy chain constant region to immunoglobulin Fc receptors on immune cells^8,9^.

Although all different licensed formulations of IVIG contain pooled IgG, there are differences in their manufacturing and content, which may result in different outcomes^10^. Enzyme processing (β-propiolactonation) is believed to confer a higher risk of IVIG-resistance^11,12^. Preservation in acid conditions (acidification) is prone to development of acute coronary abnormalities^12^. Moreover, major determinants of IVIG components, including concentration, volume, osmolality, sodium, and sugar content, may also play roles in therapeutic efficacy^13,14^. In recent years, due to limited availability, IVIG preparations used in Taiwan have mainly comprised two brands: TBSF and Privigen. Since 2008, IVIG manufactured by CSL Behring Australia from domestic blood donors obtained by Taiwan Blood Services Foundation (TBSF) has dominated the local market based on a national policy of self-sufficiency in blood-derived therapies^15^. Then in 2017, another IVIG preparation, Privigen (CSL Behring AG, Bern, Switzerland), which was prepared using foreign blood donors, was introduced. Although both IVIG have a similar liquid form, with high IgG levels as well as minimal IgA and IgM levels, there are certain differences between them, such as blood source, IgG concentration, and stabilizers. However, a head-to-head comparison of these two products from the same manufacturing group has never been conducted.

The aim of the study was to conduct a head to head comparison of two IVIGs, TBSF and Privigen, from a nationwide perspective.

## Materials and methods

### Data sources

Taiwan’s National Health Insurance (NHI) is a compulsory social welfare program that was launched in 1995, with a current coverage rate of 99.99% of Taiwan’s population. The National Health Insurance Research Database (NHIRD) consists of medical reimbursement claims in the NHI program and is managed by the National Health Research Institutes in Taiwan^16,17^. All researchers are required to conduct on-site analysis to protect the confidentiality of patients or care providers when they retrieve the detailed medical information^18^. The dataset used for our study consisted of inpatient expenditures by admission (DD files), details of inpatient orders (DO files), details of ambulatory care orders (OO files), and ambulatory care expenditure by visit (CD files) from the NHIRD.

### Study population

From January 2015 to November 2020, patients under 2 years old with a first or second admission diagnosis code of KD (International Classification of Diseases, Ninth Revision, Clinical Modification (ICD-9-CM) 446.1 and ICD-10 M303), and subsequently received treatment with intravenous IVIG (The Anatomical Therapeutic Chemical (ATC) Classification System code J06BA02) were enrolled^19^. The index date was defined as the date an individual was admitted. Congenital heart diseases were defined based on admission diagnosis codes, as follows: ICD9-CM 745–747 and ICD10 Q20–28. Patients included in our study were divided into 2 groups based on the brand of IVIG that was used: TBSF (NHI codes: KC00841248, KC00841263l; CSL Behring Australia proprietary limited) and Privigen (NHI codes: KC00965240, KC00965248, KC00965255, KC00965263; CSL Behring AG, Bern, Switzerland). Their differences are summarized in Table 1. TBSF accounted for the majority of cases in this study because it has been licensed in the domestic market since 2008. Privigen was approved for clinical use in 2017.

**Table 1:** Characteristics of two brands of intravenous immunoglobulin (IVIG).

Trade name	TBSF human immunoglobulin	Privigen human immunoglobulin
Manufacturer	CSL Behring Australia proprietary limited	CSL Behring AG, Bern, Switzerland
Blood source	pooled human plasma obtained from Taiwan	pooled human plasma supplied outside of domestic blood services arrangements
Preparation	Cold ethanol-PEG precipitation, anion exchange diethylaminoethanol (DEAE) chromatography	Cold ethanol fractionation, octanoic acid fractionation, and anion exchange chromatography
Composition		
Concentration (%)	6%	10%
Excipients	100 g/L maltose	250 mmol/L l-proline
Protein (mg/mL)	60	100
Sugar content	maltose	Sugar-free
Sodium content	0.85% at 5% concentration	Trace amount, ≤ 1 mmol/L
pH	4.25	4.6–5.0
Osmolality (mOsm/L)	Isotonic, 240 mOsm/L	Isotonic, 240 to 440 mOsm/L
IgA conc. (ug/mL)	< 25	< 25
IgM conc. (ug/mL)	trace	trace
IgG subclass		
IgG 1 (%)	61	69
IgG 2 (%)	36	26
IgG 3 (%)	3	3
IgG 4 (%)	1	2
Ig MWD	Mono + dimer > 90%	Mono + dimer > 98%
Used period	2008	2007

PEG, Polyethylene glycol fractionation; Ig MWD, IgG, Molecular weight density.

### Outcome measurements

The primary outcome was IVIG resistance, which we defined as the total amount exceeding 26 g in one admission according to a previous study and our own sensitivity analysis^12^.The previous study performed sensitivity analysis, revealing no significant changes of unresponsive IVIG cut-off point from a total dose 24 g to 38 g. We avoided 25 g as treatment failure cut-off point because the product packaging of Privigen was five grams in a unit. Based on the average weight of 12 kg for a 2-year-old child, a single dose of IVIG 2 g/kg for children with a body weight above 10 kg may have been misclassified as two courses of IVIG.

The secondary outcomes were coronary artery involvement and recurrence. Patients required long-term anti-platelet or anti-coagulation therapy if they had coronary artery aneurysm or dilatation after KD. Thus, we defined patients with coronary involvement as receiving prolonged use of aspirin (ATC code, N02BA) or anti-coagulation agents (ATC code, B01A) between 180 and 360 days after the index date. Recurrence was defined as readmission for IVIG therapy occurring more than 30 days after previous KD index day and continuing until the end of the follow-up period.

### Ethics approval and consent to participate

This study was conducted with the approval of the Institutional Review Board of Taichung Veterans General Hospital with approval number: CE14320A-8, and requirement of patients’ consent to participate was waived. All methods were conducted in compliance with the relevant guidelines and regulations.

### Statistics

Data retrieval and analysis were conducted by SAS version 9.4 (SAS Institute, Cary, North Carolina, USA), and a *P* value of < 0.05 was considered statistically significant. All quantitative data were expressed as the mean ± standard deviation (SD) and percentages as specified. Pearson’s χ2 test was applied for categorical variables and Student’s t test for continuous variables, as appropriate. For primary and secondary outcomes, the multiple logistic regression model was applied for adjusting age, gender, and congenital heart disease.

## Results

**Figure 1:**
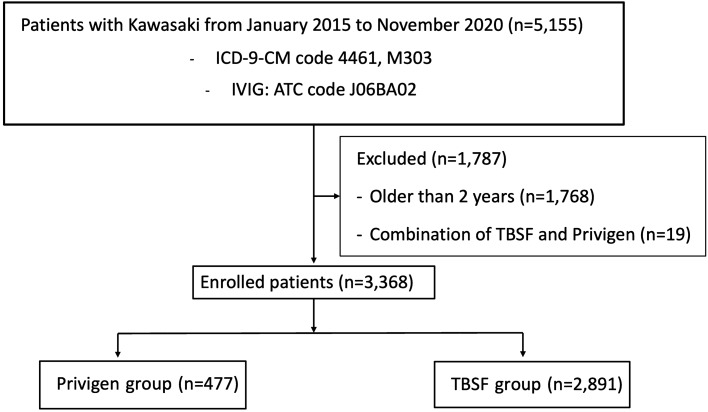
Flowchart of the patient and control selections. 5155 Kawasaki disease patients who were hospitalized under 2 years of age between January 1, 2015 to November 30, 2020 were recognized. Abbreviations: ICD-9-CM, International Classification of Diseases, Ninth Revision, Clinical Modification; IVIG, Intravenous immunoglobulin; ATC code, The Anatomical Therapeutic Chemical code.

During the study period, a total of 5155 KD patients who received IVIG therapy were identified. Among them, 1768 children were excluded by age criteria and another 19 patients were not enrolled due to combined use of TBSF and Privigen. Finally, 3368 KD children below the age of 2 years were enrolled (Fig. 1). The demographic data of patients receiving either of the two brands of IVIG are presented in Table 2. We stratified KD into three age groups: those aged less than or equal to 6 months, those aged between 6 months and 1 year, and those older than 1 year. Younger patients tended to receive TBSF, possibly attributable to the fact that each vial of TBSF contains 3 g, in contrast to Privigen vials which contain 5 g. There were widely differing case numbers in each group because Privigen had not been introduced in Taiwan until 2017. No significant differences in gender and congenital heart disease were found among patients between the two IVIG brands.

**Table 2:** Demographic data of Kawasaki disease (KD) patients receiving two different brands of immunoglobulin. Expressed as n (%).

Characteristic	TBSF group (n = 2891)	Privigen group (n = 477)	Total	*P *value
	n (%)	n (%)		
Age				0.064
≤ 6 m	466 (16.1)	77 (16.1)	543	
6–12 m	1018 (35.2)	143 (30)	1161	
> 12 m	1407 (48.7)	257 (53.9)	1664	
Gender				0.366
Female	1203 (41.6)	188 (39.4)	1391	
Male	1688 (58.4)	289 (60.6)	1977	
Year				< 0.001
2015	508 (17.6)	0 (0)	508	
2016	650 (22.5)	0 (0)	650	
2017	600 (20.8)	21 (4.4)	621	
2018	481 (16.6)	101 (21.2)	582	
2019	376 (13)	172 (36.1)	548	
2020	276 (9.5)	183 (38.4)	459	
Congenital heart disease				0.936
No	2621 (90.7)	433 (90.8)	3054	
Yes	270 (9.3)	44 (9.2)	314	

### Primary outcome and secondary outcomes

Outcomes are summarized in Table 3. Privigen demonstrated a lower IVIG resistance rate at 9.4% in comparison to TBSF, which exhibited a rate of 9.7% (odds ratio 0.72, 95% CI 0.52–0.99). Patients who received Privigen therapy had a lower odds of coronary artery involvement. The odds ratio was 0.38 (95% CI 0.18–0.82, *p* = 0.014). There is no difference in recurrence rate (odds ratio 0.60, 95% CI 0.22–1.68). This may have been caused by limited statistical power due to very few cases of recurrence.

**Table 3:** Results of multiple logistic regression models for primary and secondary outcomes.

Outcomes	TBSF group (n = 2891)	Privigen group (n = 477)	OR	95%CI	*P *value
	n (%)	n (%)			
Primary outcome					
IVIG resistance	280 (9.7)	45 (9.4)	0.72	0.52–0.99	0.041
Secondary outcomes					
Coronary artery involvement	109 (3.8)	7 (1.5)	0.38	0.18–0.82	0.014
Recurrence	43 (1.5)	4 (0.8)	0.60	0.22–1.68	0.332

Expressed as n (%), OR, Odds ratio; CI, Confidence interval; IVIG, Intravenous immunoglobulin; TBSF, Taiwan Blood Services Foundation.

### Sensitivity analysis for primary outcomes

We defined a total dose exceeding 26 g as a surrogate endpoint for IVIG resistance. To test the robustness of this surrogate endpoint, we performed a sensitivity analysis, as shown in Table 4. There were no significant differences when we changed the endpoint definition from total amount exceeding 26 to 28 gm in one admission.

**Table 4:** Sensitivity analysis of definition for primary outcomes.

Primary outcomes	TBSF group (n = 2891)	Privigen group (n = 477)	OR	95%CI	*P *value
	n (%)	n (%)			
Cutoff points (gm)					
Total IVIG exceed 26 gm	280 (9.7)	45 (9.4)	0.72	0.52–0.99	0.041
Total IVIG exceed 27 gm	280 (9.7)	45 (9.4)	0.72	0.52–0.99	0.041
Total IVIG exceed 28 gm	214 (7.4)	45 (9.4)	0.92	0.66–1.28	0.625

Expressed as n (%), OR, Odds ratio; CI, Confidence interval; IVIG, Intravenous immunoglobulin; TBSF, Taiwan Blood Services Foundation.

### Sensitivity analysis for primary and secondary outcomes in different recruitment years

In our study, it is worth noting that all cases predating the year 2017 were subjected to treatment with TBSF, as the utilization of Privigen only became prevalent from 2017 onward. Therefore, the potential for bias stemming from the disparity in recruitment years between the TBSF group (2015–2020) and the Privigen group (2017–2020) was a concern. We conducted a sensitivity analysis of primary and secondary outcomes to elucidate the years 2015–2020, 2017–2020 and 2018–2020, as presented in Table 5. The primary outcome and secondary outcomes did not change significantly when changing the beginning year.

**Table 5:** Sensitivity analysis of multiple logistic regression models for primary and secondary outcomes in recruitment years of 2015–2020, 2017–2020 and 2018–2020.

Characteristic	Year	TBSF group	Privigen group	OR	95%CI	*P *value
		n (%)	n (%)			
Primary outcome						
IVIG resistance	2015–2020	280 (9.7)	45 (9.4)	0.72	0.52–0.99	0.041
	2017–2020	164 (9.5)	45 (9.4)	0.73	0.52–1.02	0.062
	2018–2020	114 (10.1)	40 (8.8)	0.58	0.40–0.84	0.004
Secondary outcome						
Coronary artery involvement	2015–2020	109 (3.8)	7 (1.5)	0.38	0.18–0.82	0.041
	2017–2020	56 (3.2)	7 (1.5)	0.43	0.20–0.95	0.036
	2018–2020	37 (3.3)	7 (1.5)	0.43	0.19–0.96	0.039
Recurrence	2015–2020	43 (1.5)	4 (0.8)	0.60	0.22–1.68	0.332
	2017–2020	16 (0.9)	4 (0.8)	0.95	0.32–2.83	0.920
	2018–2020	7 (0.6)	4 (0.9)	1.47	0.43–5.02	0.541

Expressed as n (%), OR, Odds ratio; CI, Confidence interval; IVIG, Intravenous immunoglobulin; TBSF, Taiwan Blood Services Foundation.

## Discussion

This longitudinal cohort is a large nationwide comparative effectiveness study for IVIG (Privigen and TBSF) in the acute phase of KD. In this study, we conduct a head to head comparison of two IVIG, TBSF and Privigen. Privigen contributed to a lower IVIG resistance rate and reduced coronary artery involvement comparing to TBSF. KD recurrence did not differ significantly. The result showed Privigen might possibly offer better clinical outcome in acute KD.

Numerous studies have demonstrated the vital role of IVIG replacement therapy and immunomodulation for primary immunodeficiency diseases (PID), idiopathic thrombocytopenic purpura (ITP), kawasaki disease (KD), and allogeneic bone marrow transplantation. Owing to its activity in the regulation of the immune system, it has clinical utility in a range of autoimmune and inflammatory diseases, even as an off-label adjunctive therapy in patients with coronavirus disease 2019 (COVID-19) infection^20–22^. A 10-year population study conducted in Taiwan from 2008 to 2017 showed that KD accounted for the top two indications (20.5%) among the total distribution of IVIG utility^15^. This highlights the importance of IVIG in the treatment of KD. We found a possible explanation for the different therapeutic outcomes in our study may lie in its distinctive composition, which can be affected by the blood source (domestic versus foreign), concentration, stabilizers, and the proportion of IgG molecular weight density.

TBSF derived from Taiwanese plasma was desirable for the treatment of patients infected with Enterovirus 71^23^. It can be inferred that pathogen neutralization titers of IVIG derived from domestic plasma have an established ability to protect individuals against endemic infections. Meanwhile, Privigen is an imported IVIG product. In our study, TBSF was not superior in its effectiveness as a treatment for KD despite the fact that it was produced using domestic blood sources. This indicates that the etiology of KD may involve a level of complexity that is more commonly seen in autoimmune diseases^24,25^.

Commercial IVIG products are available at concentrations of 3%, 5%, 6%, 9%, 10%, and 12%. For initial treatment in elderly patients or in those who may be at risk of renal failure, a lower concentration of infusion should be chosen^26^. The infusion volume of TBSF required for an equivalent dose of IVIG is greater than that of Privigen. Manlhiot and colleagues reported a 10% IVIG concentration provided a shorter infusion time and may lead to better clinical outcomes, including shorter duration of fever after IVIG infusion and less IVIG unresponsiveness compared to a 5% IVIG concentration^27^. In Taiwan, most physicians use IVIG rapid infusion for 12 h and give a second dose of IVIG 48 h after the first dose if refractory. Thus, the difference in infusion time by different concentration cannot explain the phenomenon we observed in this study. A small retrospective cohort and another nationwide inpatient database study in Japan observed no significant difference between 5 and 10% IVIG for the treatment of patients with acute-phase KD^28,29^. Based on aforementioned, we therefore infer that the difference in concentration of TBSF and Privigen is not responsible for clinical differences.

The biological mechanisms of IVIG in the treatment of KD are unclear. There are several hypotheses. It has been postulated that the Fc portion of the immunoglobulin G molecule play a therapeutic role in KD through immune complex neutralization, opsonic activity enhancement, and complement modulation^8^. During the manufacturing process of IVIG, anything that will affect Fc portion of the IgG molecule may attribute to different clinical outcomes. The superior beneficial therapeutic effect observed in Privigen in our study could be possibly attributed to the stabilization methods, and preservation of the more efficient binding affinity of monomeric IgG to Fc gamma receptors. Privigen is purified by a combination of cold ethanol fractionation, octanoic acid fractionation, and anion exchange chromatography. It is stabilized with natural amphiphilic amino acid l-proline, which can optimize intermediate pH 4.8–5.3 to minimize the IgG dimer aggregation, up to 30% lower, in solutions compared with other stabilizer excipients^30^. It has been shown that the presence of dimeric and multimeric IgGs in IVIG products is nonetheless indispensable for the inhibitory effect on Fc gamma receptors-mediated phagocytosis and its blockage of the inflammatory pathway^31^. Since the formation of idiotype-anti-idiotype dimers is a reversible process, the presence of an amphiphilic stabilizer, such as l-proline, was effective in restricting the formation of IgG dimers and preserving the immunomodulatory reactivity of anti-idiotypic antibodies. On the other hand, TBSF undergoes purification via anion exchange diethylaminoethanol (DEAE) chromatography and Macro-Prep High Q Resin support to eliminate immunoglobulin aggregates and is stabilized using sugar content. TBSF contains 10% maltose as a stabilizer to reduce IgG aggregation and is suspended at pH 4.25^32^. In a previous review by Dantal et al., the sugar content affected the osmolarity and was associated with increased significant renal risks of failure, insufficiency, and osmotic nephrosis^20,26^. Also, the presence of IgG dimers and IgG aggregation in IVIG may be associated with vasoactive adverse reactions, such as lowered blood pressure, headache, fever, and flushing during intravenous infusion^33^. The different stabilization methods may account for the better clinical outcomes in our study. l-proline, used as a stabilizer in Privigen, provided the optimal conditions for stability and restricted IgG dimer formation, which could explain the superior clinical outcomes.

There are four subclasses of IgG in humans: IgG1, IgG2, IgG3, and IgG4. The predominant isotype is IgG1, accounting for more than 60% of the total IgG in human serum^34^. Each subclass has a distinct susceptibility with regard to antibody-mediated immune response^35^. Typically, IgG1 and IgG3 are potent triggers of effector mechanisms and interact efficiently with most Fc gamma receptors, whereas IgG2 and IgG4 show reduced affinity and induce more subtle responses^36^. As for the proportion of IgG1 plus IgG3 and the total amount of IgG molecular weight density, these values are higher in Privigen than in TBSF. These differences might confer a therapeutic effect and account for the better clinical outcomes in our study.

The enzymatic process of β-propiolactonation is believed to be associated with an elevated risk of IVIG resistance. This poorer immunomodulation function in comparison with the ion exchange procedure is ascribed to the modifications occurring in the Fc portion of immunoglobulin during the alkylation and acylation of proteins in β-propiolactonation^37,38^. It is noteworthy that TBSF undergoes purification through anion exchange diethylaminoethanol (DEAE) chromatography, while Privigen is purified through a combination of cold ethanol fractionation, octanoic acid fractionation, and anion exchange chromatography^39^. Neither of these purification processes involves β-propiolactonation.

Preservation in acid conditions (acidification) is prone to increase the risk of coronary aneurysms because a substantial infusion of acidic fluids may lead to a reduction in intracellular tone and an increase in vascular tension^27,40^. The suspension of TBSF at pH 4.25 may offer a potential explanation for the rise in acute coronary aneurysms in our study.

KD primarily affects children younger than 5 years of age, and the peak incidence occurred from 6 months to 2 years of age, which includes more than 50% of all KD patients^41^. We limited the range of the patients’ ages from 6 months to 2 years to reduce the misclassification of claims data, as body weight could greatly vary in children older than 2 years. In previous observations by Lin et al.,^12^ administration of total dose exceeding 24 gm to 38 gm did not change significantly in sensitivity study and the findings are consistent with our own sensitivity study. In our own sensitivity analysis, as presented in Table 4, it was observed that a total dose exceeding 28 g did not attain statistical significance. It may suggest that 28 g as cutoff point is excessively high, potentially resulting in an insufficient sample size for achieving adequate statistical power. We avoid utilizing the treatment failure cutoff points of 25 g and 27 g due to the packaging specifications of TBSF, which comprises three grams per unit, and Privigen, which contains five grams per unit, both being multiples of three and five, respectively. Also, in line with the previous literature, IVIG resistance rates were around 10%^42^. Privigen and TBSF had IVIG resistance rates of 9.4% and 9.7%, respectively, in our study, indicating that 26 gm may be reasonable as the IVIG unresponsive cut-off point. In our study, IVIG resistance displayed significant differences between two commercial IVIG. In Taiwan, the majority of physicians discontinued the use of aspirin after 6 weeks in patients with normal coronary arteries, according to AHA and Japanese consensus guidelines^43^. In patients with an aneurysm, regardless of its size, aspirin would continue to be prescribed. For those with a persistent medium-sized aneurysm, anticoagulation may be considered. We defined prolonged use of antiplatelets or anticoagulants 180 to 360 days after the index date in our study as an indicator of possible coronary artery involvement. In our study, Privigen reduced prolonged anti-platelets or anti-coagulants use indicating decreased occurrence of coronary artery aneurysms in long-term follow-up and may help to prevent thrombus formation. For recurrence, no significant differences were found owing to insufficient sample size limiting statistical power.

Potential limitations inherent to this retrospective design should be noted. The large-scale, population-based database (NHIRD) used in this study does not contain information on certain variables, such as medical history, physical examination, laboratory testing, defervescence, duration of hospital stay, and electrocardiogram cardiac assessments. The severity of cardiac condition (coronary artery Z-score or absolute dimension) could not be identified from the diagnosis codes alone. IVIG resistance was defined by a total dose exceeding 26 g which may cause misclassification affected by weight variation. Disease severity and the subsequent treatment, which could potentially impact outcomes, such as steroid therapy, biological products including infliximab, ulinastatin therapy, immunosuppressive agent cyclosporine, and plasma exchange remain undisclosed in our study. There are also no laboratory data in the NHIRD. Therefore, high-risk populations cannot be identified.

## Conclusion

**Figure 2:**
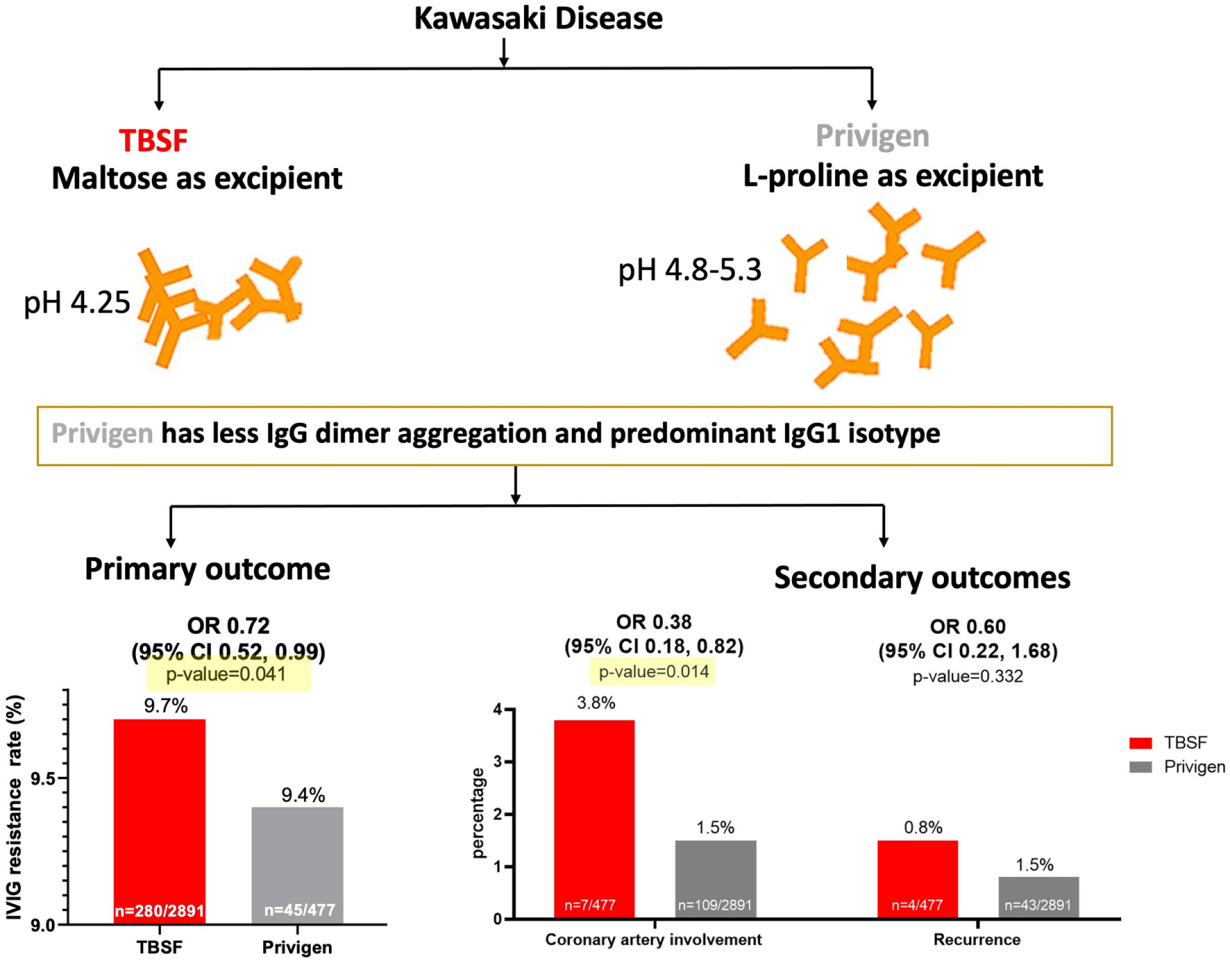
Based on our study's findings, we have concluded that Privigen may exhibit a lower incidence of IVIG resistance and reduced coronary artery involvement. This disparity may be attributed to the presence of l-proline as Privigen excipient, which optimize intermediate pH 4.8–5.3 to minimize the IgG dimer aggregation. Also, Privigen has higher proportion of IgG1 isotype, known for its potent triggering of effector mechanisms and efficient interaction with most Fc gamma receptors.

Privigen might have a lower rate of IVIG resistance and reduced coronary artery involvement. This discrepancy might due to the concentration, the stabilizer, or the source of plasma, all of which serve to minimize IgG dimer aggregation and provide a higher proportion of IgG1 to interact with Fc gamma receptors as Fig. 2. Further investigation is needed to compare the effectiveness of different IVIGs in the large randomized controlled clinical trial.

## Data Availability

The datasets presented in this article are not readily available because data release is not allowed by the National Health Insurance Research Database. Requests to access the datasets should be directed to Dr. Chien-Heng Lin/ epid@ms39.hinet.net.

## Abbreviations

IVIG: Intravenous immunoglobulin
IgG: Immunoglobulin G
FC: Fractional change
TBSF: Taiwan Blood Services Foundation
NHIRD: National Health Insurance Research Database
KD: Kawasaki disease
